# Assessment of Selected Structural Properties of High-Speed Friction Welded Joints Made of Unalloyed Structural Steel

**DOI:** 10.3390/ma16010093

**Published:** 2022-12-22

**Authors:** Beata Skowrońska, Tomasz Chmielewski, Dariusz Zasada

**Affiliations:** 1 Faculty of Mechanical and Industrial Engineering, Institute of Manufacturing Technologies, Warsaw University of Technology, Narbutta 85, 02-524 Warsaw, Poland; 2 Faculty of Advanced Technologies and Chemistry, Military University of Technology, Kaliskiego 2, 00-908 Warsaw, Poland

**Keywords:** S235JR structural steel, solid-state welding, rotary friction welding

## Abstract

Commonly used S235JR structural steel, generally associated with good weldability, was joined by high-speed friction welding (HSFW). The friction welding tests were performed with a rotational speed of n = 8000 rpm and four different values of the unit pressure in the friction phase (p_f_) in the range of 64–255 MPa. The obtained joints were subjected to metallographic observations using an optical microscope; in selected zones of friction joints the average grain size was specified in accordance with the EN ISO 643:2012 standard; the hardness of friction joints was measured using the Vickers method. The friction-welded joint with the highest p_f_ was EBSD-investigated. The obtained friction-welded joints resembled an hourglass, and the microstructure of individual zones of the joints differed depending on the height (axis, radius) of the observations. The generated joining conditions resulted in a significant refinement of the microstructure in the friction weld—the average grain size is about 1 µm^2^ (for base material it was 21 µm^2^). The highest increase in hardness above 340 HV0.1 was recorded in the friction weld of the welded joint with the lowest used value pressure in the friction phase. Such a sharp increase in hardness can make the resulting friction-welded joint become sensitive to dynamic or fatigue loads. The electron backscatter diffraction (EBSD) investigation confirmed the strong refinement of the microstructure in the friction-welded joint and the occurrence of the phenomenon of dynamic recrystallization (DRX). The friction weld was also characterized by a large share of high-angle boundaries (HAGBs) >80%. These results may indicate that during high-speed friction welding it is possible to create conditions like those obtained during the High-Pressure Torsion (the method used to produce UFG materials) process.

## 1. Introduction

Unalloyed ferritic-pearlitic structural steel is considered to be a material with good weldability; however, in extreme welding conditions its joints are, like other metals, exposed to the risk of defects. While, in fusion welding conditions, the danger of significant hardening and brittleness of the joint results from exceeding the critical cooling speed in the temperature range from 800 to 500 °C, the classic rotary friction welding of S235JR steel does not generate a high risk of defective joints.

Friction welding is a solid-state joining method where the same materials are joined without the liquid phase (below the melting point) [1]. In the rotary friction welding process, the source of energy needed for joining is the kinetic friction energy converted into heat on the friction surface directly in the area of the joint being formed [2]. Using the process parameters i.e., rotational speed [3] and friction time [4], it is possible to precisely measure the amount of heat released [5]. Among the friction welding methods in industry, rotary welding is the most commonly used, where at least one of the elements connected is a rotary solid [6]. In addition, high-speed rotary friction welding (HSFW) is used more and more often in the industry, which, due to the speed of >5000 rpm, allows the best obtaining of a joint in a relatively short time (100 ms ÷ 2 s) [7]. The set speed influences, among other things, the intensity of heating of the materials to be joined and thus the change in thickness of the plastically deformed layer and the rate of shortening of the components, as well as the change in thermal power of the friction process [8,9]. It is these features of the HSFW process that positively influences the quality of joints made of advanced materials [10]. In the case of materials sensitive to the joining heat, such as UFG materials, the weakening of the friction joints (caused by the degradation of the microstructure of the base material) was limited [11,12,13], and in addition good quality joints were obtained for materials such as: alloy EN-AC-44200 with Al/Al_2_O_3_ composite materials [14], stainless steel with free-cutting steel [15], tungsten with mild steel [16], NiAl and FeAl alloys with St3S steel [17] and titanium alloys [18]. On the other hand, such a dynamic action on the material causes a high degree of deformation and significant strengthening due to grain refinement, and excessive work hardening in the friction zone may have a negative impact on the operational properties of the joint.

In this paper, the widely used S235JR structural steel was subjected to high-speed friction welding to verify the potential of using advanced joining methods for typical construction materials.

## 2. Materials and Methods

**Figure 1:**
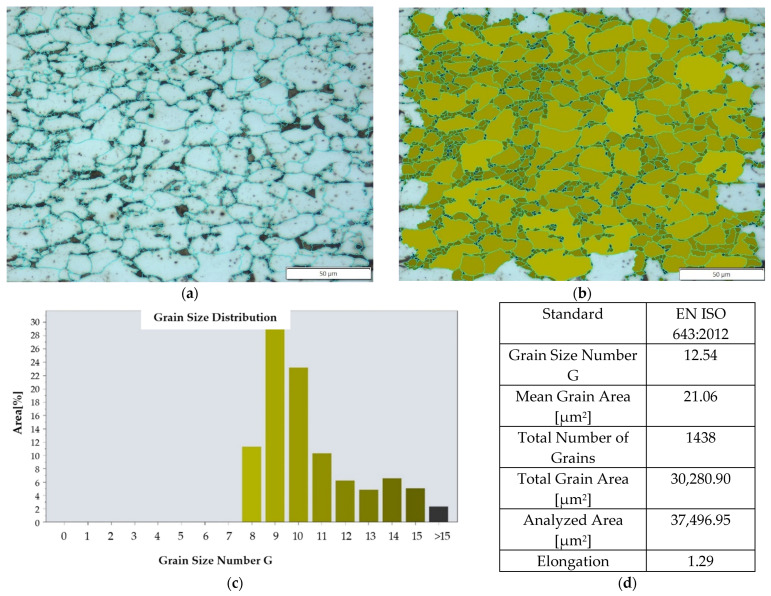
Grain size measurement (made on the optical microscope) of the base material, a sample taken parallel to the rod axis: (**a**) grain outline, (**b**) grain size map, (**c**) grain size histogram, (**d**) result for the examined area.

Rods made of S235JR steel with a diameter of 6 mm and a length of 30 mm were subjected to high-speed friction welding. The base material (BM) according to the manufacturer’s specification was characterized by the following strength properties, UTS = 621 MPa and YS = 552 MPa, and the chemical composition of the material is presented in Table 1. The tested steel is characterized by a ferritic-pearlitic microstructure with an average grain size in accordance with EN ISO 643:2012 at the level of G13, which corresponds to an area of 21 µm^2^, Figure 1.

**Figure 2:**
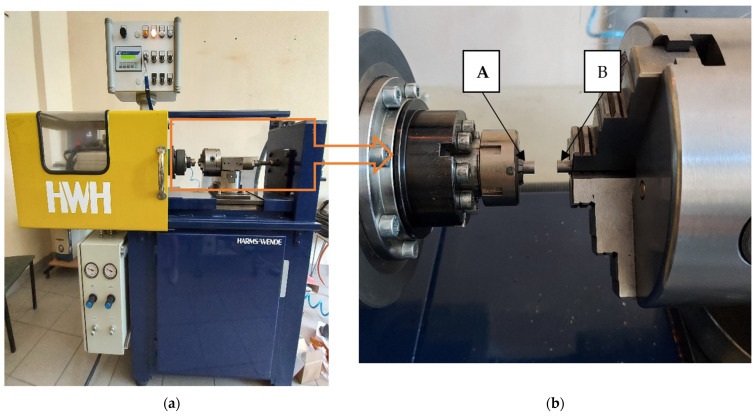
(**a**) High-speed rotary friction machine—general view, (**b**) fixing of specimens: A—rotating sample, B—stationary sample.

Tests of high-speed friction welding were carried out on the Harms & Wende RSM210 (Hamburg, Germany) friction welder (Figure 2a). The precise control system of the welding machine enables the setting of the spindle speed in the range of 6000 ÷ 24,000 rpm and the friction time in milliseconds. In this way, it is possible to create “rapid” friction welding conditions unattainable with conventional friction welding.

S235JR steel was friction welded using the following parameters: rotational speed n = 8000 rpm, friction time t_f_ = 60 ms, pressure on the face of welded counter-samples (p_f_) in the range 95.5–245.5 MPa. Within a single friction welding cycle, the pressure values in the friction and upsetting phases were at the same level. Fixing was carried out on the specimens with a 10 mm distance between the faces of the rotating specimen (A) and the stationary specimen (B) (Figure 2b). The process parameters were selected on the basis of optimization according to the criteria of the highest rotational speed, the narrowest HAZ and the shortest welding time.

Metallographic observations and hardness measurements were carried out on samples taken perpendicular to the plane of friction, along the axis of the rods. First, metallographic observations were carried out on an Olympus BX51M optical microscope coupled with the Olympus Stream Essentials software for qualitative and quantitative analysis of the microstructures studied (including measurement of grain size according to EN ISO 643:2012). The samples were placed in a thermosetting resin on a laboratory press—ATM-OPAL-410. Preparation of the friction joints included wet grinding procedures with the use of 80 to 2500 grit sandpaper and polishing with Al_2_O_3_ suspension with 1 µm gradation. For metallographic observations using Nomar—Differential Interference Contrast (DIC), the samples were polished on colloidal suspension of silica (OP-S). The final step in the preparation of the friction welded samples was etching with 5% Nital.

The Vickers hardness measurements were carried out on a LEITZ MINILOAD 8375 hardness tester at a load of 0.981 N and an impact time of the indenter on the material of 15 s. They were performed on etched samples to determine the center of the weld.

The EBSD-investigation was performed using a Quanta 3D FEG high-resolution scanning electron microscope equipped with, e.g., an electron backscattered diffraction (EBSD) analysis system. Preparation of the sample for testing consisted of grinding on silicon carbide abrasive papers with a grain size of 400 to 4000. The last stage was polishing with a diamond suspension with a grain size of 3 and 0.25 µm and a SiO_2_ suspension with a grain size of 0.1 µm.

## 3. Results and Discussion

### 3.1. Metallographic Investigation

**Figure 3:**
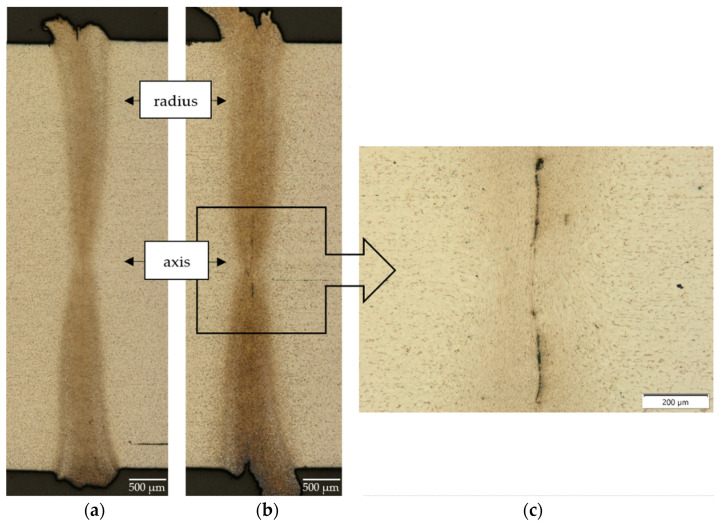
Macrostructures of high-speed friction-welded joints (**a**) with p_f_ = 191 MPa, (**b**) with p_f_ = 127.3 MPa; (**c**) Microstructure of the observed discontinuity.

Figure 3 shows the results of metallographic observations made on an optical microscope of selected high-speed friction-welded joints. The obtained friction-welded joints resemble an hourglass shape. In the joint, with unit pressure in the friction phase on the faces of the samples of 127.3 MPa, discontinuity (imperfection) of the joint was observed in the area of the axis (around which the rotation of the moving sample is performed) (Figure 3c). The joint realized under pressure of 191 MPa (Figure 3a) is sound, continuous and free of any defects and the friction weld is homogeneous with no possibility of distinguising between the original friction welding plane. In the remaining friction-welded joints, no discontinuities or other imperfections typical of welded joints i.e., pores, gas bubbles or non-metallic inclusions, are revealed [19,20].

**Figure 4:**
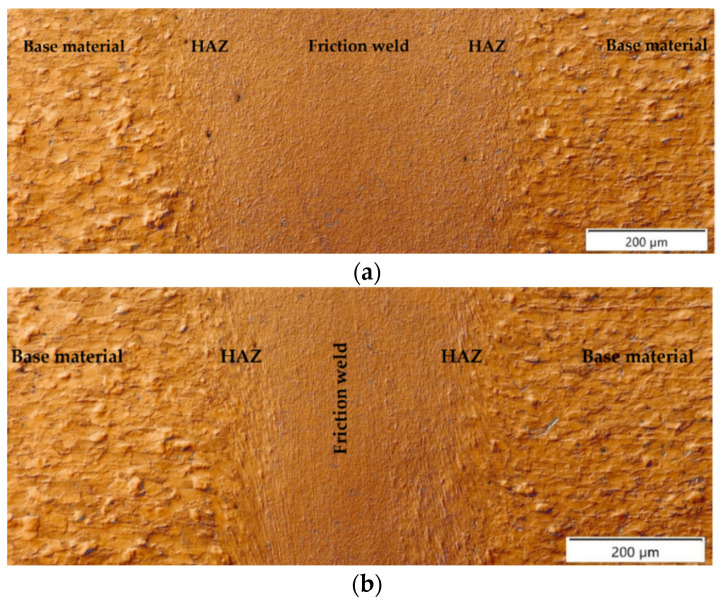
Microstructure of a high-speed friction welded joint with p_f_ = 191 MPa made: (**a**) on the radius, (**b**) at the axis, (optical observations with DIC).

**Figure 5:**
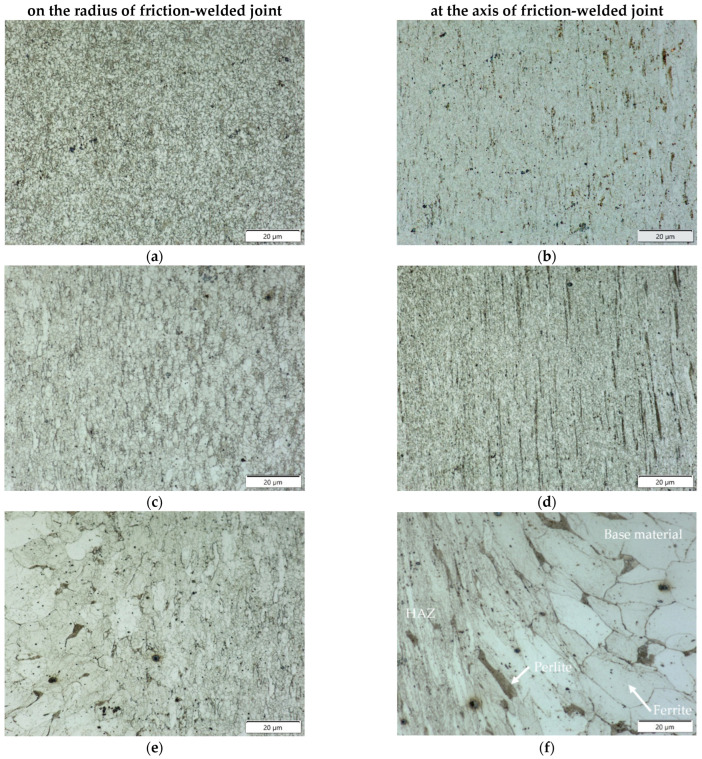
Microstructure of a friction-welded joint with p_f_ = 191 MPa: (**a**,**b**) in friction weld, (**c**,**d**) in HAZ, (**e**,**f**) in transition zone between HAZ and BM.

The conditions arising during high-speed friction welding affect not only the width of the joint, but also the different nature of the microstructural changes in the heat-affected zone (HAZ)—these differences are particularly evident in the axis and on the radius (marked in Figure 3) of friction-welded joints. Figure 4 shows panoramic photographs of the microstructure of the joint cross-section taken in the areas mentioned above. The microstructure of the individual zones (friction weld, HAZ, BM) of the examined areas of the friction-welded joint are shown in Figure 5.

In all the described cases, the joint’s width at the radius (close to the external surface) is approximately twice as large as at the axis. However, accurate measurement of the joint and the HAZ width is impossible due to the lack of a clear boundary between them.

The microstructure of high-speed rotary friction joints of mild steel is heterogeneous. In the base material, we observe a two-phase ferritic-pearlitic structure with equiaxial grains of both phases, partially visible on the right-hand side of Figure 5f. The microstructure changes towards the joint; closer to the joint we observe the plastic deformation and defragmentation of the grains and their movement towards the flash of the weld, Figure 5e,f. In the HAZ and area of large plastic deformation (Figure 5c,d) significant grain refinement was rreveale; however, this is different in the zone close to the joint axis and on the radius (close to the external surface) of the friction-welded rods. The direction of flow of the material is clearly visible in both zones. Figure 5a,b show the microstructure of the joint in the friction zone where the largest grain refinement takes place, with a clear directivity in the joint axis and no directionality on the radius close to the external surface. The descriptive differences in the microstructure are the result of a significant difference in the linear velocity of friction close to the axis and on the radius of the rods.

**Figure 6:**
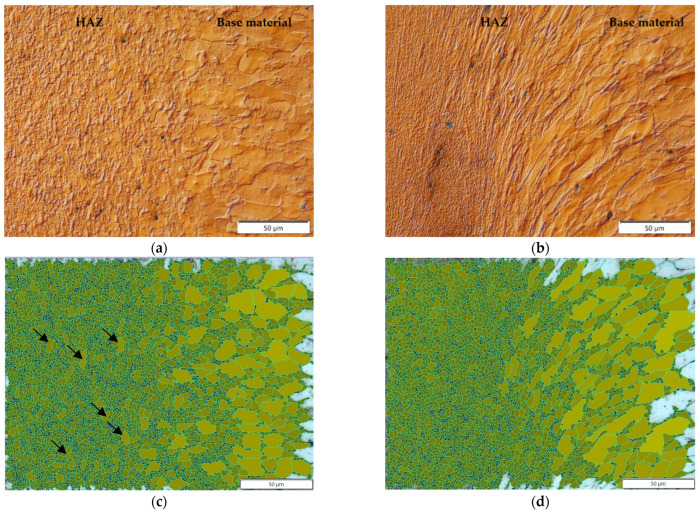
Microstructure of the HAZ of a high-speed friction-welded joint (observations with DIC): (**a**) on the radius, (**b**) at the axis; (**c**,**d**) grain size measurement corresponding to the microstructure from points a and b (measurement area: 220 µm × 160 µm).

In the area of the axis, strongly defragmented grains of base material were observed aligned along the friction weld in the direction of extrusion of the plasticized material into the flash. On both sides of the axis of rotation of the rods, bands of deformed material point in the radius direction; similar deformed material behavior was observed in austenitic steel joints with UFG microstructure [12]. This situation and distribution of specific properties on the radius is strongly related with the function of heat distribution with variable friction speed on the radius. In contrast, in the HAZ zone at the radius, no strongly deformed grains of the base material were observed, only their gradual fragmentation. Measurement of the grain size in the HAZ revealed, a uniformly refined microstructure of the material at axis and single, with larger grains (corresponding to G 10 according to EN ISO 643:2012) occurring in the HAZ at the radius (indicated by arrows in Figure 6).

**Figure 7:**
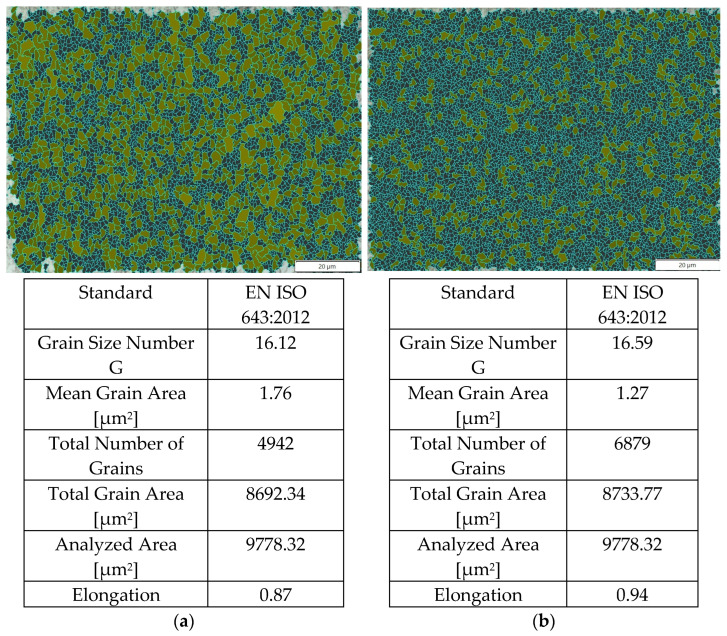
Measurement of grain size on the radius of the friction-welded joint with p_f_ = 191 MPa: (**a**) HAZ, (**b**) friction weld.

Due to the fact that a very narrow high-speed friction-welded joint was obtained on the axis, measurement of the grain size so that the field of observation covered the microstructure of one zone was only possible on the radius of the friction-welded joints. Figure 7 shows the result of measurements taken in the HAZ and in the friction weld (at 1000× magnification, the measurement area is 120 µm × 100 µm). The highest degree of refinement of the microstructure was obtained in the friction weld, where the average grain size was 1.27 µm^2^ (corresponding to G 17). In the HAZ, there was also a high degree of grain refinement to a level of—1.76 µm^2^.

The strong refinement of the grains in the friction weld and HAZ is a result of the friction generated between the joined surfaces, which also causes the grains to shear into each other. On the other hand, the observed differences in the width of the joints and in the degree of deformation of the microstructure are due, among other things, to differences in the amount of heat released on the radius and the way the material is heated and cooled in both areas of the friction joint (at the axis and at the radius). Given the linear velocity distribution, theoretically the greatest amount of heat should be released at the periphery of the rods to be joined. However, the authors of the papers [21,22] showed that the applied frictional pressure is not uniformly distributed over the contact surface and that the maximum heat release occurs in the 1/2 ÷ 2/3R range. The difference in heat distribution in the near-surface layer of rotary friction-welded rods is additionally due to the occurrence of radial and convective heat. On the other hand, an increase in the temperature at the interface also causes changes in the friction moment and, consequently, changes in the friction conditions and the intensity of heat generated [23].

### 3.2. Hardness

**Figure 8:**
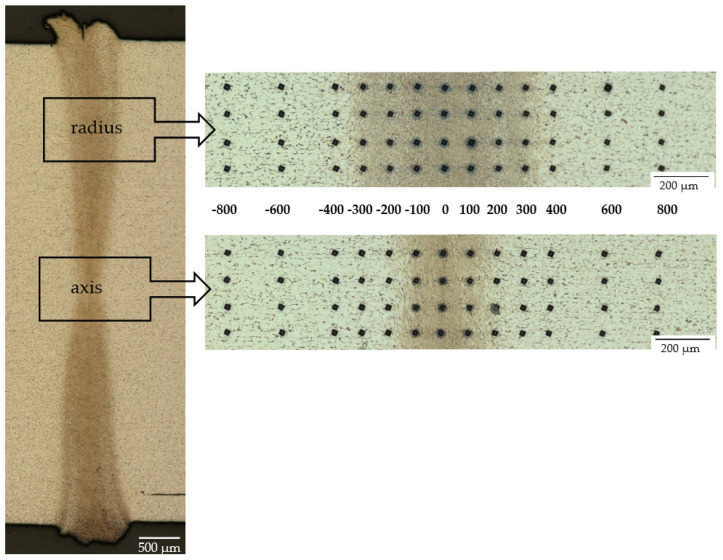
An example of the distribution of measuring points.

**Figure 9:**
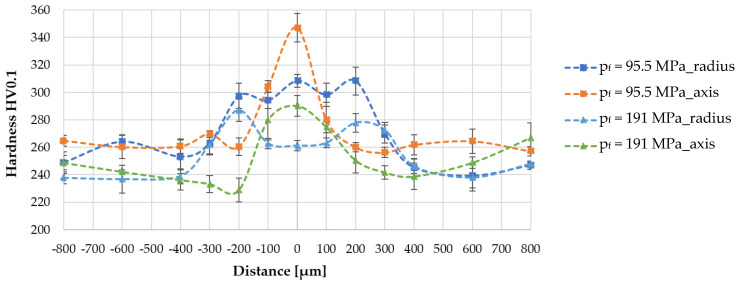
Hardness distribution of high-speed friction welded joints made of S2335JR steel.

Due to the differences in the microstructure of the high-speed friction-welded joints observed during metallographic testing, hardness measurements were carried out in two areas (Figure 8)—in the axis of the specimens and at the radius (1 mm from the edge of the rods, i.e., 2/3 R). Measurements were started from the center of the friction weld (point 0) and on both sides of the friction weld; 4 measurement points were taken at 100 µm intervals and 2 measurement points at 200 µm intervals (in the base material). To obtain statistically reliable results, measurements were made in 4 measurement lines (spaced at 100–150 µm intervals), Figure 8. Each value shown on the hardness distribution is the mean value of the 4 measurements together with the standard deviation rods (t-student with 95% confidence level). The hardness distributions obtained for selected high-speed friction welded joints are shown in Figure 9.

Analysis of the results presented in Figure 9 showed that the joint areas tested (on axis and radius) differed not only in microstructure, but also in the shape of the hardness distribution courses and the maximum value obtained. Higher hardness was registered at the joint axis. There was a sharp increase in hardness—up to 347 HV0.1, in the friction welded joint with a lower unit pressure. In contrast, the hardness distribution at the radius of this joint has several hardness peaks oscillating near 300 HV0.1. Doubling the unit pressure in the friction phase (to 191 MPa) resulted in a softening of the hardness distribution at the axis and the maximum value was 290 HV0.1. At the radius in the friction weld, the hardness was 20 HV0.1, lower than that of the HAZ. This result indicates that friction welding conditions can be obtained that lead to a normalized structure in the material to be joined. Such high hardness values were not obtained in joints welded at 1000–2000 rpm [24].

### 3.3. EBSD-Investigation

The effect of high-speed friction welding conditions on the degree of microstructure change (grain size, shape, disorientation, and degree of grains deformation) of S235JR steel in the joint was evaluated by electron microscopy. EBSD-investigation were carried out at a height of ½ the radius of the rods, i.e., 1.5 mm from the surface of the rods, where, according to the actual heat distribution, the most stable conditions should be [21,25].

The tests began with the determination of the center of the weld (point ‘0′), which is “contractual” due to the lack of clear boundaries between the individual zones of the joint. Then 4 measurements were made in 100 µm steps and 1 measurement in the base material. When selecting the magnification, it was considered that the field of observation was as large as possible—to characterize the microstructure from as large an area as possible without losing the representativeness of the results obtained. For each measurement area, the field of view is 25 µm × 25 µm and contains more than 300 grains (for the base material, 100 grains).

The EBSD characteristics of the welded joint with unit pressure in the friction phase of 254.7 MPa were made based on the analysis: Inverse Pole Figure (IPF) Map with high-angle grain boundaries (HAGBs), Grain Size (GS) and Mistorientation Angle (MA). On the other hand, the quantitative assessment of the processes of microstructure reconstruction (i.e., recrystallization) was performed based on the Grain Orientation Spread (GOS) parameter [26]. In this case, the deformed grains show a higher mean value of local disorientation than the recrystallized grains, for which the GOS parameter <2° was adopted.

**Figure 10:**
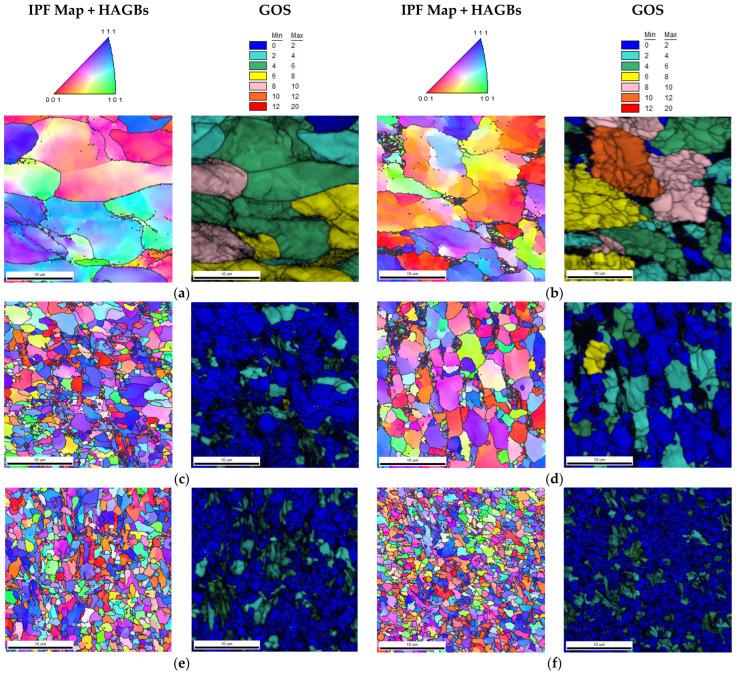
Inverse pole figure (IPF) maps with HAGBs, and GOS parameter images of measurement areas of a high-speed friction welded joint made of S235JR steel; (**a**) Base material; (**b**) ‘400′; (**c**) ‘300′; (**d**) ‘200′; (**e**) ‘100′; (**f**) ‘0′–Friction weld.

**Figure 11:**
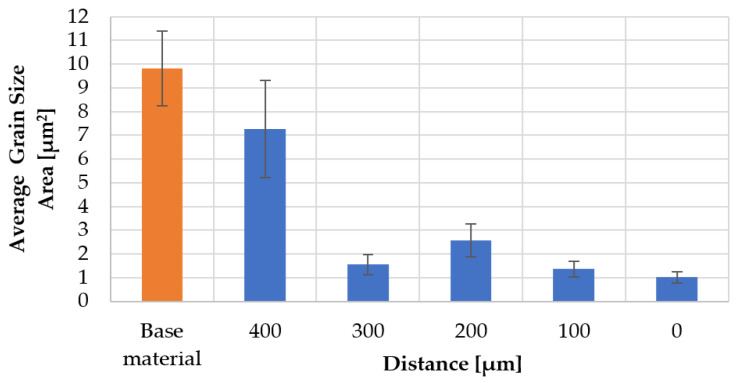
Distribution of the mean grain size obtained in the measurement areas.

**Figure 12:**
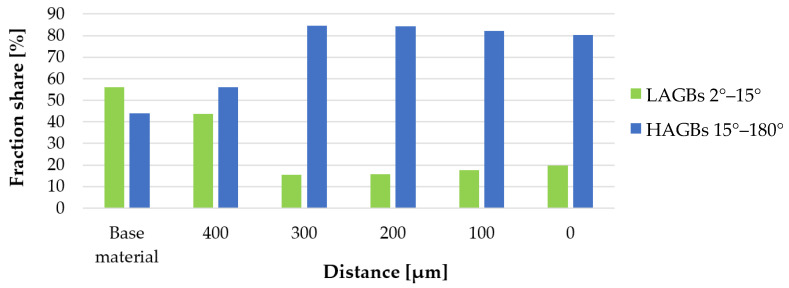
Share of low- and high-angle grain boundary fractions in the measurement areas.

**Figure 13:**
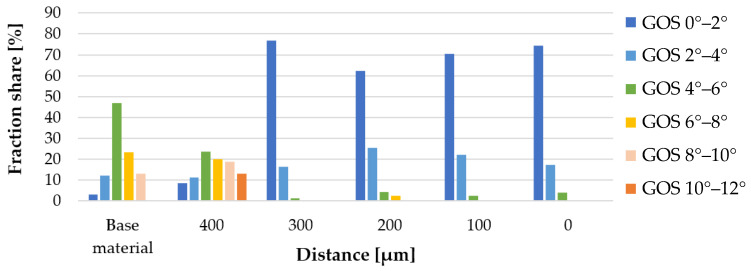
Distribution of the GOS parameter fraction in the measurement areas.

The IPF-map and GOS images obtained for the individual measurement areas are shown in Figure 10. The results of the mean grain size and the share of low- (<15°) (LAGBs) and high-angle (>15°) grain boundaries corresponding to the images in Figure 10 are presented in the graphs, respectively Figure 11 and Figure 12. The distribution of the GOS parameter in individual measurement areas is shown in Figure 13.

The friction occurring in the plane of joining of the friction-welded rods resulted in a strong refinement and recrystallization of the microstructure in the joint (Figure 10). The highest degree of grain refinement, down to the level of 1 µm^2^, was obtained in the friction weld (point ‘0′). As one moves away from the friction weld, the mean grain size increases (Figure 11), while for the measuring area 300 µm away, the microstructure refinement was again recorded at the level of 1.5 µm^2^. In the measurement areas ‘0’–’300’, the proportion of HAGs was almost twice as high as in the base material—and amounted to min. 80% (Figure 12). In these areas the share of recrystallized grains was equally high, >60% (Figure 13). At the same time in points “0” and “300”, it exceeded 74%. In the measurement area 400 µm away from the friction weld, the microstructure consists of large, deformed grains, as in the base material, while in Figure 10b the effect of the supplied heat and areas of fine recrystallized grains can also be observed.

The crystal structure of the friction weld is shaped as a result of heat-activated physical processes in a high-gradient stress field [27] e.g., diffusion in a solid [28], grain shear and the creation of new boundaries [29], with dynamic recrystallization (DRX) in the area of energy supply [30,31].

## 4. Conclusions

The results of preliminary tests of high-speed friction welding of S235JR structural steel have shown that:

- it is possible to obtain correct joints in a very short time (60 ms). The obtained friction joints resembled an hourglass, therefore the tests carried out at different radius of the friction joint (in the axis and on the radius) showed differences in the degree of deformation and refinement of the microstructure of individual zones of the friction joint. Measurement of the grain size according to EN ISO 643: 2012 (for an observation field of 120 µm × 100 µm) showed grain refinement in the friction weld to an average of 1.27 µm^2^, and in HAZ 1.76 µm^2^. The average grain size of the base material was 21 µm^2^. The analysis of hardness distributions showed that these areas (at the axis and on the radius) also differ in the degree of strengthening of the microstructure. In the extreme case, the hardness of the friction weld increased above 340 HV0.1. Too high an increase in the hardness of the joint may cause its sensitive to dynamic or fatigue loads.
- The results of the EBSD-investigation (for an observation field of 25 µm × 25 µm) confirmed the strong refinement of the average grain size in the friction joint up to 300 µm from the friction plane. The microstructure in this area is also characterized by a high share (>80%) of high-angle grain boundaries and a share of recrystallized grains at the level of 70%. The results show that during high-speed friction welding, the phenomenon of dynamic recrystallization took place, but also that it is possible to obtain conditions like those existing during High Pressure Torsion (the method used to produce UFG materials). At the same time, as they moved away from the friction weld, the average grain size increased, and the share of the recrystallized grains fraction decreased. And at 300 µm from the center of the friction weld, another decrease in the average grain size, observation on the optical microscope also showed the occurrence of normalization.
- The width of the zone where the friction welding cycle causes changes in microstructure and hardness is comparable. Hardness distribution is not closely related to grain size distribution. Grain size and reorientation of the crystals are not the only factors shaping hardness. The character of the thermal cycle of friction welding and the gradient temperature distribution are of great importance.
- The task with a very short time of friction <1 s and high values of rotational speed and unit pressure in the friction phase resulted in the creation of a sharp gradient of microstructural properties of the joint. Such severe conditions of friction welding generated significant changes in the material properties in the joint zone.

## Figures and Tables

**Table 1:** Chemical composition (wt.%) of S235JR steel (manufacturer specification).

C	Si	Mn	P	S	N	Cu	Fe
0.069	0.170	0.510	0.0100	0.0250	0.0075	0.230	balance

## Data Availability

Experimental methods and results are available from the authors.
